# Supplementary material for: Community engagement in health services research on elimination of lymphatic filariasis: A systematic review
Source: PLOS Glob Public Health. 2023 Jan 17;3(1):e0001226. doi: 10.1371/journal.pgph.0001226 (PMC10021320; doi:10.1371/journal.pgph.0001226)
Supplement: S1 Text — (DOC) [file pgph.0001226.s002.doc]

**S1 Text. R commands for mapping Figure 2**

library(rworldmap)

## Welcome to rworldmap ###

For a short introduction type : vignette('rworldmap')

Warning message:

package ‘rworldmap’ was built under R version 4.1.3

map= read.csv( file.choose())

map

Country Number

1 Papua New Guinea 1

2 India 11

3 Thailand 1

4 Myanmar 2

5 Indonesia 1

sPDF <- joinCountryData2Map(map,joinCode = "NAME",nameJoinColumn = "Country",verbose = TRUE)

5 codes from your data successfully matched countries in the map

0 codes from your data failed to match with a country code in the map

failedCodes failedCountries

238 codes from the map weren't represented in your data

mapCountryData(sPDF,nameColumnToPlot="Country",colourPalette="rainbow",catMethod="categorical",oceanCol= "lightblue", mapRegion=c("asia" , "Papua New Guinea"))

**Data source (excel file save as .csv)**

| Country | Number |
| --- | --- |
| Papua New Guinea | 1 |
| India | 11 |
| Thailand | 1 |
| Myanmar | 2 |
| Indonesia | 1 |
