# Supplementary material for: Community engagement in health services research on elimination of lymphatic filariasis: A systematic review
Source: PLOS Glob Public Health. 2023 Jan 17;3(1):e0001226. doi: 10.1371/journal.pgph.0001226 (PMC10021320; doi:10.1371/journal.pgph.0001226)
Supplement: S1 Table — (DOC) [file pgph.0001226.s003.doc]

**S1 Table**

**Search strategy**

| Database | Search strategy | Results |
| --- | --- | --- |
| Ovid Full text | 1 community.mp. [mp=title, abstract, full text, caption text]  2 community engagement.mp. [mp=title, abstract, full text, caption text]  3 participatory.mp. [mp=title, abstract, full text, caption text]  4 action research.mp. [mp=title, abstract, full text, caption text]  5 participatory research.mp. [mp=title, abstract, full text, caption text]  6 participatory action research.mp. [mp=title, abstract, full text, caption text]  7 community-based research.mp. [mp=title, abstract, full text, caption text]  8 action science.mp. [mp=title, abstract, full text, caption text]  9 action inquiry.mp. [mp=title, abstract, full text, caption text]  10 cooperative inquiry.mp. [mp=title, abstract, full text, caption text]  11 health service research.mp. [mp=title, abstract, full text, caption text]  12 filariasis$.mp. [mp=title, abstract, full text, caption text]  1 or 2 or 3 or 4 or 5 or 6 or 7 or 8 or 9 or 10  11 and 12 | 56 |
| Ovid Medline | 1. community.mp. or exp Residence Characteristics/ 2. Community-Based Participatory Research/ or Community Participation/ or community engagement.mp. or Health Promotion/ 3. participatory.mp. or Health Services Research/ or Community Participation/ 4. action research.mp. or exp Health Services Research/ 5. Community-Based Participatory Research/ or participatory research.mp. 6. participatory action research.mp. or Qualitative Research/ 7. Community Health Services/ or community-based research.mp. 8. action science.mp. 9. Cooperative Behavior/ or "Delivery of Health Care"/ or action inquiry.mp. 10. exp Health Services Research/ or cooperative inquiry.mp. 11. 1 or 2 or 3 or 4 or 5 or 6 or 7 or 8 or 9 or 10 12. filariasis.mp. or exp Filariasis/ 13. 11 and 12 | 1166 |
| PubMed | (((((((((((community) OR (community engagement)) OR (participatory)) OR (action research)) OR (participatory research)) OR (participatory action research)) OR (community-based research)) OR (action science)) OR (action inquiry)) OR (cooperative inquiry))) AND (filariasis) | 1988 |
| PubMed Central | Search ((((((((((community[MeSH Terms]) OR community engagement[MeSH Terms]) OR participatory[MeSH Terms]) OR action research[MeSH Terms]) OR participatory action research[MeSH Terms]) OR cooperative inquiry[MeSH Terms]) OR action inquiry[MeSH Terms]) OR community-based research[MeSH Terms]) OR participatory action research[MeSH Terms])) AND filariasis[MeSH Terms] | 31 |
| The Cochrane library | (Community):ti,ab,kw OR (community engagement):ti,ab,kw OR (participatory):ti,ab,kw OR (action research):ti,ab,kw  (filariasis)  #1 AND #2 | 78 |

**Web of Science:**

TOPIC: (community OR "community engagement" OR " community-based research " OR " participatory ") AND (hsr OR " health service research" OR "action research") AND (LF OR “filariasis” OR “lymphatic filariasis” OR “elephantiasis”)
