# Supplementary material for: Community engagement in health services research on elimination of lymphatic filariasis: A systematic review
Source: PLOS Glob Public Health. 2023 Jan 17;3(1):e0001226. doi: 10.1371/journal.pgph.0001226 (PMC10021320; doi:10.1371/journal.pgph.0001226)
Supplement: S2 Table — (DOC) [file pgph.0001226.s004.doc]

**Supplementary Table 2. Excluded studies**

| No. | Author, yr | Reason for exclusion | Citation |  |
| --- | --- | --- | --- | --- |
| 1 | Ramaiah, 2006 | Not addressed CE | Ramaiah KD, Das PK, Vanamail P, et al. The impact of six rounds of single-dose mass administration of diethylcarbamazine or ivermectin on the transmission of Wuchereria bancrofti by Culex quinquefasciatus and its implications for lymphatic filariasis elimination programmes. Trop Med Int Health. 2003 ;8(12):1082-92. |  |
| 2 | Krentel, 2021 | Narrative review | Krentel A, Gyapongc M, McFarland D et al. Keeping communities at the centre of efforts to eliminate lymphatic filariasis: learning from the past to reach a future free of lymphatic filariasis. International Health 2021; 13, Suppl.1: S55–S59 |  |
| 3 | Well, 2019 | Clinical trial for drug efficacy | Weil GJ, Bogus J, Christian M, et al. The safety of double- and triple-drug community mass drug administration for lymphatic filariasis: A multicenter, open-label, cluster-randomized study. PLoS Med 2019, 16(6): e1002839 |  |
| 4 | Kapa, 2021 | Narrative review | Kapa DR, Mohamed A. Progress and impact of 20 years of a lymphatic filariasis elimination programme in South-East Asia. International Health 2021; 13, Suppl.1: S17–S21 |  |
| 5 | Leang, 2004 | Not address CE | Leang R, Socheat D, Bin B, Bunkea T, et al Assessment of disease and infection of lymphatic filariasis in Northeastern Cambodia. Tropical Medicine & International Health. 2004; 9(10):1115–20. |  |
| 6 | Win, 2018 | post-MDA coverage surveys | Win KM, Tripathy JP, Maung TM, et al. Rapid progress towards elimination of lymphatic filariasis in endemic regions of Myanmar as a result of 16 years of anti-filarial activities (2001– 2016). Tropical medicine and health. 2018; 46(1):14. |  |
| 7 | Toothong,2015 | surveys | Toothong, T., Tipayamongkholgul, M., Suwannapong, N. *et al.* Evaluation of mass drug administration in the program to control imported lymphatic filariasis in Thailand. *BMC Public Health* 2015, 15, 975. |  |
| 8 | Zinia,2011 | surveys | Zinia T. Nujum, Coverage and compliance to mass drug administration for lymphatic filariasis elimination in a district of Kerala, India, International Health, 2011, 3: 22–26, |  |
| 9 | Panicker, 1982 | Health education campaign | Panicker KN, Dhanda V (‎1992)‎. Community participation in the control of filariasis *World health forum 1992 ; 13(‎2/3)‎ : 177-181*<https://apps.who.int/iris/handle/10665/51900> |  |
| 10 | Hussain,2014 | Not CE context | Hussain, M.A., Sitha, A.K., Swain, S. *et al.* Mass drug administration for lymphatic filariasis elimination in a coastal state of India: a study on barriers to coverage and compliance. *Infect Dis Poverty* **3,**31 (2014) |  |
| 11 | Kumar,2009 | Not CE context | Kumar A, Kumar P, Nagaraj K, Nayak D, Ashok L, Ashok K. A study on coverage and compliance of mass drug administration programme for elimination of filariasis in Udupi district, Karnataka, India. J Vector Borne Dis. 2009 Sep;46(3):237-40. |  |
| 12 | Babu, 2008 | Not CE context | Babu BV, Mishra S. Mass drug administration under the programme to eliminate lymphatic filariasis in Orissa, India: a mixed-methods study to identify factors associated with compliance and non-compliance, Transactions of The Royal Society of Tropical Medicine and Hygiene, 2008; 102: 1207–1213, |  |
| 13 | Rao,2018 | Not CE/CP context | Rao RU, Samarasekera SD, Nagodavithana KC, et al.. Comprehensive assessment of a hotspot with persistent bancroftian filariasis in Coastal Sri Lanka. Am J Trop Med Hyg. 2018 ;99(3):735-742. |  |
| 14 | Krental, 2012 | Not CE/CP context | Krentel A, Aunger R. Causal chain mapping: a novel method to analyse treatment compliance decisions relating to lymphatic filariasis elimination in Alor, Indonesia. Health Policy Plan. 2012;27(5):384-95 |  |
| 15 | Burkot, 2006 | A narrative review | Burkot TR, Durrheim DN, Melrose WD, et al The argument for integrating vector control with multiple drug administration campaigns to ensure elimination of lymphatic filariasis. Filaria J. 2006 16;5:10. |  |
| 16 | Narahari, 2013 | Not CE/CP context | Narahari SR, Bose KS, Aggithaya MG, et al Community level morbidity control of lymphoedema using self-care and integrative treatment in two lymphatic filariasis endemic districts of South India: a non-randomized interventional study. Trans R Soc Trop Med Hyg 2013; 107: 566−77. |  |
| 17 | Sunish, 2015 | Not CE/CP context | Sunish IP, Kalimuthu M, Rajendran R, et al. Decline in lymphatic filariasis transmission with annual mass drug administration using DEC with and without albendazole over a 10year period in India. Parasitol Int. 2015 ;64(1):1-4. |  |

CE: community engagement CP: community participation;
