# Supplementary material for: Community engagement in health services research on elimination of lymphatic filariasis: A systematic review
Source: PLOS Glob Public Health. 2023 Jan 17;3(1):e0001226. doi: 10.1371/journal.pgph.0001226 (PMC10021320; doi:10.1371/journal.pgph.0001226)
Supplement: S3 Table — (DOC) [file pgph.0001226.s005.doc]

S3 Table. Summary of risk of bias assessment using ROBINS-1 tool

| N4o. | Study | Confounding bias | Selection bias | Classification bias | Bias due to deviations from intended interventions | Bias due to missing data | Outcome measurement bias^a^ | Reporting bias^a^ | Overall bias |
| --- | --- | --- | --- | --- | --- | --- | --- | --- | --- |
| 1 | Aggithaya,2013 | Moderate | Low | Low | Moderate | Low | Low | Low | Moderate |
| 2 | Aye, 2018 | Moderate | Low | Low | Low | Low | Low | Low | Moderate |
| 3 | Babu, 2004 | Moderate | Low | Low | Moderate | Low | Low | Low | Moderate |
| 4 | Babu, 2006 | Moderate | Low | Low | Moderate | Low | Low | Low | Moderate |
| 5 | Dickson,2018 | Moderate | Low | Low | Moderate | Low | Low | Moderate | Moderate |
| 6 | Krentel, 2016 | Moderate | Low | Low | Low | Low | Low | Low | Moderate |
| 7 | Lahariya,2008 | Moderate | Low | Low | Moderate | Low | Low | Low | Moderate |
| 8 | Nandha, 2007 | Moderate | Low | Low | Moderate | Low | Low | Low | Moderate |
| 9 | Nandha, 2012 | Moderate | Low | Low | Moderate | Low | Low | Low | Moderate |
| 10 | Patel, 2012 | Moderate | Low | Low | Low | Low | Low | Low | Moderate |
| 11 | Ramaiah,2000 | Moderate | Low | Low | Moderate | Low | Low | Low | Moderate |
| 12 | Ramaiah, 2001 | Moderate | Low | Low | Moderate | Low | Low | Low | Moderate |
| 13 | Rajendran, 2010 | Moderate | Serious | Moderate | Moderate | Moderate | Moderate | Low | Serious |
| 14 | Rojanapanus,  2019 | Moderate | Low | Low | Moderate | Low | Low | Moderate | Moderate |
| 15 | Sunish, 2016 | Moderate | Low | Low | Moderate | Low | Low | Low | Moderate |
| 16 | Wynd,2007 | Not enough information | Not enough information | Not enough information | Not enough information | Not enough information | Not enough information | Not enough information | Not enough information |
