# Supplementary material for: Community engagement in health services research on elimination of lymphatic filariasis: A systematic review
Source: PLOS Glob Public Health. 2023 Jan 17;3(1):e0001226. doi: 10.1371/journal.pgph.0001226 (PMC10021320; doi:10.1371/journal.pgph.0001226)
Supplement: S4 Table — (DOC) [file pgph.0001226.s006.doc]

**S4 Table**

**Barriers to community engagement in HSR**

| **Study, yr [ ref#]** | Lack of engagement of  participating bodies | Lack of interest & dependency | Lack of enthusiasm | Workload | Limited budget/ resources | Misconcepts | Staffing | social group mobilization | Appropriate technology  challenges | Social challenges | Geographical challenge | transportation |
| --- | --- | --- | --- | --- | --- | --- | --- | --- | --- | --- | --- | --- |
| Ramaiah (2001)  [32] | √ | √ | √ | √ |  |  | √ |  |  |  |  |  |
| Babu (2006)  [24] | √ |  |  |  |  | √ |  |  | √ |  |  |  |
| Aggithaya (2013)[21] |  |  |  |  | √ |  |  |  |  |  | √ |  |
| Krentel  (2016) [26] |  |  |  |  |  |  |  |  | √ |  |  |  |
| Patel (2012) [30] |  |  |  |  |  | √ |  |  |  |  |  |  |
| Babu (2003) [23] | √ |  | √ |  |  |  |  |  | √ | √ |  |  |

**Facilitators to community engagement in HSR**

| **Study, yr**  **[ref#]** | active engagement of  participating bodies | interest & dependency | enthusiasm | Workload | Adequate budget/resources | Sufficient staffing | social group mobilization | Appropriate technology facilitation | Good social interactions |
| --- | --- | --- | --- | --- | --- | --- | --- | --- | --- |
| Ramaiah (2001)  [32] |  |  |  |  |  |  | √ | √ | √ |
| Babu (2006)  [24] |  |  |  |  |  |  |  | √ | √ |
| Aye (2018)  [22] | √ |  |  |  |  |  |  | √ |  |
| Rajendran (2010)  [33] |  |  |  |  |  |  |  | √ |  |
| Aggithaya (2013)  [21] | √ |  |  |  |  |  |  |  |  |
