# Supplementary material for: Community engagement in health services research on elimination of lymphatic filariasis: A systematic review
Source: PLOS Glob Public Health. 2023 Jan 17;3(1):e0001226. doi: 10.1371/journal.pgph.0001226 (PMC10021320; doi:10.1371/journal.pgph.0001226)
Supplement: Attachment — Submitted filename: Our reply_.doc [file pgph.0001226.s007.doc]

Q/

We've checked your submission and before we can proceed, we need you to address the following issues:
Figure 2: please (a) provide a direct link to the base layer of the map used and ensure this is also included in the figure legend; (b) provide a link to the terms of use / license information for the base layer. We cannot publish proprietary or copyrighted maps (e.g. Google Maps, Mapquest) and the terms of use for your map base layer must be compatible with our CC-BY 4.0 license.

**Our reply/**

Figure 2 is prepared for this review by ourselves, and it is not a copy from a published/unpublished source. We used R package (*rworldmap)* for this illustration. For clarity and necessary management, we have provided the commands in Supplementary file. Thank you

**In the revised version**

**Data synthesis**

**Line #160-163**

*“For a map indicating the geographic distribution of studies identified, we use R®version (4.11) pertinent to “rworldmap” package. The data used for mapping and commands are provided in S1 document.”*

**Supplementary Information**

*“S 1 document: R commands for mapping Figure 2”*
