# Supplementary material for: Community engagement in health services research on elimination of lymphatic filariasis: A systematic review
Source: PLOS Glob Public Health. 2023 Jan 17;3(1):e0001226. doi: 10.1371/journal.pgph.0001226 (PMC10021320; doi:10.1371/journal.pgph.0001226)
Supplement: Attachment — Submitted filename: our reply_CN_HHA_MAW.doc [file pgph.0001226.s008.doc]

PGPH-D-22-01335
Community Engagement in Health Services Research on Elimination of Lymphatic Filariasis: A Systematic Review
PLOS Global Public Health

Journal Requirements:

Q/

1. Please review your reference list to ensure that it is complete and correct. If you have cited papers that have been retracted, please include the rationale for doing so in the manuscript text, or remove these references and replace them with relevant current references. Any changes to the reference list should be mentioned in the rebuttal letter that accompanies your revised manuscript. If you need to cite a retracted article, indicate the article’s retracted status in the References list and also include a citation and full reference for the retraction notice.

**Our reply/**

We have checked it and there are no retracted papers in the citation.

Reference list is complete and correct.

Thank you

Q/

2. Please amend your detailed Financial Disclosure statement. This is published with the article. It must therefore be completed in full sentences and contain the exact wording you wish to be published.

a. State the initials, alongside each funding source, of each author to receive each grant.

If you did not receive any funding for this study, please simply state: “The authors received no specific funding for this work.”

**Our reply/**

We have updated, accordingly.

Thank you

**In the revised text**

**Financial Disclosure statement**

This work received financial support from TDR (the Special Programme for Research and Training in Tropical Diseases, co-sponsored by UNICEF, UNDP, the World Bank and WHO) [Project ID AP21-00287]. CN, MAW, NHH, and HHA received this award. None of the authors received salary or other funding from commercial companies. None of these authors work at commercial companies. The funders had no role in study design, data collection and analysis, decision to publish, or preparation of the manuscript.

Q/

3. We do not publish any copyright or trademark symbols that usually accompany proprietary names, eg (R), (C), or TM  (e.g. next to drug or reagent names). Please remove all instances of trademark/copyright symbols throughout the text, including ® on pages 9.

**Our reply/**

We have updated, accordingly.

Thank you

**In the revised text**

**On page #9 at line # 163-164**

For a map indicating the geographic distribution of studies identified, we use *R*version (4.11) pertinent to “*rworldmap”* package.

**<<<<<<<<<<<<<<<<<<<<<<<<<<<<<<<<<<<<<<<<<<<<<<<<<<<<<<<<<<<<<<**

**Reviewer #1**: The authors did a good work on the systematic review. Please find my concerns as comments:
**Q/**

**Abstract:**
1. The last sentence reads incomplete. Are "Future well-designed prospective studies..." recommended?

**Our reply/**

We have updated, accordingly.

Thank you

**In the revised text**

**Abstract**

**Line # 37-39**

Future well-designed prospective studies with attention to the description of mechanisms of engagement, facilitating the whole process and reporting the community level outcome are recommended.

Q/

**Introduction:**
1. Line 68, write out CE at first instance, or insert acronym on Line 65 if it means "Community Engagement.
**Our reply/**

We have spell out CE, and then made an abbreviation.

Thank you

**In the revised text**

**On page #3-=4 at line # 68-69**

From the health services perspective on the research context, studies reported that community engagement (CE) strengthened the conduct of research [8,9], and was a core value in participatory health research [10].

Q/

Methods:
1. Authors listed some databases searched for articles, but I could not find details in the supplementary S1 table such as for Web of Science, Science Direct, Google scholar, WHO library. Kindly clarify.
**Our reply/**

We have added details of the strategy used for Web of Science. For Science Direct, Google scholar, and WHO library, we have used only keywords with appropriate Bolen operators, as described in the text.

Thank you

**In the revised text**

**Supplementary Table 1**

**Web of Science:**

TOPIC: (community OR "community engagement" OR " community-based research " OR " participatory ") AND (hsr OR " health service research" OR "action research") AND (LF OR “filariasis” OR “lymphatic filariasis” OR “elephantiasis”)

2. Was there a justification or cited standardization for categorizing overall rating as high (score 4-5), moderate (score 3) and low (score ≤ 2)? Why, for example, was moderate not (score 3-2) and low (score 1-0)?

**Our reply/**

We have taken the suggested cut-off level, which is more meaningful, and updated the sum score. As indicated, we have adapted this from a published review (reference #12) with necessary modification

Thank you

**In the revised text**

**Methods**

**Assessment of the level of community engagement in HSR**

**On page #8 at line # 130-138**

We give 1 (+) score to ‘leading’ or ‘collaborating’, while 0 scores for the remaining three attributes, as described elsewhere [12]. Hence, the highest score that a study can be achieved is five. As an example, study AA showed CE as ‘consultation’ at ‘developing ideas’, collaboration at ‘developing methodology’, and ‘informed’ at data collection/ analysis, but ‘not sure’ at reporting writing and ‘collaboration’ at dissemination stage,) achieved a total score of 2 (i.e., 0+ 1+ 0+0++1= 2). To determine the extent of CE, the level of engagement across all aspects of the study was summed up and the extent determined as high extent (score 4-5), moderate extent (score 3-2) and low extent (score 0-1).

**Results**

**On page # 15 at line # 214-219**

The extent of CE in various phases of HSR was directly or indirectly measured in a variety of ways across the studies. Overall, 25% of studies (4/16, 25%) [ 20.24,26,29] were at the low level of engagement in the research context lacking involvement at the stage of ‘developing ideas’ (i.e., research planning stage) and ‘report writing’. The vast majority [21-23,25, 27,28,30, 31-35] (12/16,75%) were at the ‘moderate level’ of engagement in research context mainly by ‘collaboration’ in ‘developing methodology’ ‘collaboration’ in data collection and ‘collaboration’ for ‘dissemination of findings’ (Table 3).

**Table 3. Various forms of community and extent of engagement (N =16 studies)**

| No | Study, yr | Forms of community | | | | | | Extent of engagement in HSR | | | | | Extent of engagement | |
| --- | --- | --- | --- | --- | --- | --- | --- | --- | --- | --- | --- | --- | --- | --- |
|  |  | A | B | C | D | E | F | Developing ideas | Developing  methodology | Data collection/  analysis | Report writing | dissemination | Scoring | level |
| 1 | Aggithaya, 2013 [20] | √ |  |  |  |  | √ |  |  |  |  |  | 0+0+0+0+1=1 | Low |
| 2 | Aye,  2018 [21] | √ |  |  |  |  |  |  |  |  |  |  | 0+1+1+0+1=3 | Moderate |
| 3 | Babu,  2004 [22] | √ |  |  |  |  |  |  |  |  |  |  | 0+1+0+0+1=2 | Moderate |
| 4 | Babu,  2006 [23] | √ |  |  |  |  |  |  |  |  |  |  | 0+1+1+0+1=3 | Moderate |
| 5 | Dickson,  2018 [24] |  |  | √ | √ |  |  |  |  |  |  |  | 0+1+0+0+0=1 | Low |
| 6 | Krentel,  2016 [25] | √ |  |  |  |  |  |  |  |  |  |  | 0+1+0+0+1=2 | Moderate |
| 7 | Lahariya,  2008 [26] | √ |  |  |  |  |  |  |  |  |  |  | 0+1+0+0+0=1 | Low |
| 8 | Nandha,  2007 [27] | √ |  |  |  |  |  |  |  |  |  |  | 0+1+0+0+1=2 | Moderate |
| 9 | Narahari,  2013 [28] | √ |  |  |  |  |  |  |  |  |  |  | 0+1+0+0+1=2 | Moderate |
| 10 | Patel,  2012 [29] | √ |  | √ |  |  | √ |  |  |  |  |  | 0+0+0+0+1=1 | Low |
| 11 | Ramaiah,  2000 [30] |  |  | √ |  |  |  |  |  |  |  |  | 0+1+0+0+1=2 | Moderate |
| 12 | Ramaiah,  2001 [31] | √ |  |  |  |  |  |  |  |  |  |  | 0+0+1+0+1=2 | Moderate |
| 13 | Rajendran, 2010 [32] | √ |  |  |  |  |  |  |  |  |  |  | 0+1+1+0+1=3 | Moderate |
| 14 | Rojanapanus,  2019 [33] | √ |  | √ |  |  |  |  |  |  |  |  | 0+1+0+0+1=2 | Moderate |
| 15 | Sunish,  2016 [34] |  |  | √ |  |  |  |  |  |  |  |  | 0+1+1+0+1=3 | Moderate |
| 16 | Wynd,  2007 [35] | √ | √ | √ |  | √ | √ |  |  |  |  |  | 0+1+1+0+1=3 | Moderate |

Q/

3. If the investigators reviewed 'full-text copies' (line 142), should not your exclusion criteria include inaccessibility of full-text articles? Else, state that you were able to retrieve full-text copies of all the articles from your search strategy.

Our reply

We have added that full-text copies of all the articles from the search output were able to retrieved. Thank you
**In the revised text**

**Data collection**

**On Page #8 at line # 145-146**
The two investigators independently retrieved full-text copies that were deemed relevant and checked their eligibility. Full-text copies of all the articles from the search output were able to retrieved.

Q/

Results:
Looks good.
Our reply

Thank you

Q/

I would recommend line 230 - 234 be moved to discussion section. "For instance, limitations to utilizing the WHO definition of drug coverage..."
Our reply

Agreed and we have moved these sentences to the Discussion section. Thank you

**In the revised version**

**Discussion**

**On page # 19 at line # 284-286**

Moreover, limitations to utilizing the WHO definition of drug coverage [25], and dispensing of loose tablets with no labelling on a package [22] were technology-related issues that hindered community involvement in research context.

Q/

Figure 1 PRISMA flow: Records identified (1069) minus Duplicated records (143) leave Record screened (926). This is correct. Records removed for other reasons (378) disrupts this flow, because your diagram implies that 378 was removed from 1069, which was not the case. Please modify diagram to show where 378 was removed.
Our reply

We have corrected it. Thank you

**In the revised version**

Figure 1 PRISMA flow

Q/

Minors:
Write out ROBINS-I at first instance: "Risk Of Bias In Non-randomised Studies - of Interventions"
Our reply

We have spelled it out . Thank you

**In the revised version**

**On page # 7 at line # 118-119**

**Assessment of the methodological quality**

We evaluated the methodological quality of the included studies using Risk Of Bias In Non-randomised Studies - of Interventions"
(ROBINS -I) tool [18].

Q/

Did you consider "Stakeholders involvement" as keyword in your search strategy?

**Our reply/**

We have considered it initially. But the search output yield was fewer than that yielded by the terms we have actually used in our current strategy.

Thank you

Q/

**Reviewer #2: Summary**
Thank you for the opportunity I have to provide a review for this Community Engagement in Health Services Research on Elimination of Lymphatic Filariasis: A Systematic Review.
This is a good attempt by the authors to determine engagement of the community in health research and the conclusion showed there is need to adequate information of process of community engagement in these researches that were reviewed.

Title
• The title clearly depicts the research that was carried out with some focus on what is being reviewed, however the period or time of investigation is missing. I think it should be included.
**Our reply/**

We beg your pardon. We sincerely felt that period is not necessary in the title. We have indicated the time period in the study search. Thank you

Q/

• Each author’s contribution was clearly stated, and corresponding author’s contact is clear.

**Our reply/**

Thank you

Q/

Abstract
• The abstract is unstructured, and it allowed for a good read.
• The conclusion was clearly written
**Our reply/**

Thank you

Q/

Introduction
• The introduction provided a good background for the study.
• line 68: authors introduced an acronym (“CE”) that was not explained before. Please write it in full first so readers can understand what it is for.
• The objectives of the study were stated clearly.
**Our reply/**

We have spell out CE before making abbreviation. Thank you

**In the revised version**

**On page #3 at line # 68-69**

From the health services perspective on the research context, studies reported that community engagement (CE) strengthened the conduct of research [8,9], and was a core value in participatory health research [10].

Q/

Methods and Materials
• methods and materials are well explained, and it is reproducible
**Our reply/**

Thank you

Q/

Results
• The result is well written
**Our reply/**

Thank you

Q/

Discussion
• The discussion is focused on the results
**Our reply/**

Thank you

Q/

Conclusion
• Good conclusion
**Our reply/**

Thank you
