# Supplementary material for: Community engagement in health services research on elimination of lymphatic filariasis: A systematic review
Source: PLOS Glob Public Health. 2023 Jan 17;3(1):e0001226. doi: 10.1371/journal.pgph.0001226 (PMC10021320; doi:10.1371/journal.pgph.0001226)
Supplement: Attachment — Submitted filename: R2_Our Reply to the Comments_3 Nov.doc [file pgph.0001226.s009.doc]

PGPH-D-22-01335R1
**Community Engagement in Health Services Research on Elimination of Lymphatic Filariasis: A Systematic Review**
PLOS Global Public Health

Journal Requirements:

Q/

Please review your reference list to ensure that it is complete and correct. If you have cited papers that have been retracted, please include the rationale for doing so in the manuscript text, or remove these references and replace them with relevant current references. Any changes to the reference list should be mentioned in the rebuttal letter that accompanies your revised manuscript. If you need to cite a retracted article, indicate the article’s retracted status in the References list and also include a citation and full reference for the retraction notice.

**Our reply/**

The references have been examined for retractions through <http://retractiondatabase.org/RetractionSearch.aspx>. Reference lists contain no instances of papers that have been retracted. Thank you

Q/

Reviewer #2:

No new comments, all previous comments duly addressed

**Our reply/**

Thank you

Reviewer #3

Q/

Reviewer #3: The title should follow the PLOS requirements; it should be written in sentences case with its exceptions. Please revise it.

**Our reply/**

We have fixed it, accordingly. Thank you

**In the revised version**

**Community engagement in health services research on elimination of lymphatic filariasis: A systematic review**

Q/

The corresponding author should not be written in abbreviation.
**Our reply/**

We have spelled it out. Thank you

**In the revised version**

Corresponding author: Cho Naing

Email: [cho3699@gmail.com](mailto:cho3699@gmail.com)

Corresponding author: Maxine A Whittaker

Email: [maxine.whittaker@jcu.edu.au](mailto:maxine.whittaker@jcu.edu.au)

Q/

The sign for "These authors contributed equally" is not the correct sign. Please revise it.
**Our reply/**

Accordingly, we have fixed it. Thank you

**In the revised version**

**Authors and Affiliations**

Cho Naing^1¶^, Norah Htet Htet^2^, Htar Htar Aung^2^, Maxine A Whittaker^1¶^

^¶^Cho Naing, Maxine A Whittaker: Equal contributors and joint corresponding author

Q/

Line 80: After a semi colon the first word should be written in capital letter
**Our reply/**

We have corrected it, accordingly. Thank you

**In the revised version**

**Line # 78-79**

Taken together, we did a systematic review to address a question: What studies are available that have assessed the CE in HSR on elimination of lymphatic filariasis?

Q/

Line 240: Why full stop? I think you want to make a coma. please update it.
**Our reply/**

We have corrected it, accordingly. Thank you

**In the revised version**

**Line # 245-246**

Fifteen studies were conducted in the WHO SEA region, and only one study was from the Pacific region.

Q/

Line 244-245: The statement needs a coma.

**Our reply/**

We have corrected it, accordingly. Thank you

**In the revised version**

**Line # 249-250**

As there was inadequate description of CE in the original papers, there was concern over accuracy in data abstraction.

Q/

After the headings, you have started the text with out paragraph indentation. However, after the study limitation headings (line 290) you have started with the indentation.

**Our reply/**

We have corrected it, accordingly. There is no indentation of this part in this revised version. Thank you

**In the revised version**

**Study limitations**

**Line # 293-294**

**Study limitations**

This review has some limitations that should be acknowledged.

Reviewer #4

Q/

Reviewer #4: The manuscript has been impressively improved based on previous reviewer comments.

**Our reply/**

Thank you.

Q/

There are few areas for improvement which have been given below:
1. In outcome section of table 1, give operational definition of all the outcomes listed.
**Our reply/**

Taken together with a comment below, we have given these as descriptions in Supplementary Table 2.Suppl Table 2 has been relocated into the text and is now referred to as Table 2.

Thank you

**In the revised version**

**Table 2 Description of health services research in th**is review

| No. | Category | Subtype | Descriptions |
| --- | --- | --- | --- |
| 1 | Evaluating the quality of health services | 1A. Structure of health services   | facilities (e.g., hospitals, clinics, primary /health centre)  personnel  technology (e.g. checklist, diagnostic tool, treatment algorithm, drugs administered, etc) |
|  |  | 1B. Process of health services | interactions between the health care providers and patients/communities over time |
|  |  | 1C. Outcomes of health services   A) Beneficial effects:  An outcome that is statistically significant in favours the community intervention B) Self-efficacy: the belief their own abilities, specifically their ability to meet the challenges ahead of them and complete a task successfully      | - patient/community-reported health/functional status (e.g. MDA coverage, MDA compliance) - satisfaction with health status (e.g QoL) - satisfaction with services (e.g. drug distribution in MDA) - costs of health services. |

Q/

2. Lines 189-190- please briefly explain HSR typology (structure, process and outcome) in methodology section.
**Our reply/**

Referring to a comment above, we have relocated Supple Table 2 to the methodology. It becomes Table 2 in this revised version. Thank you

Q/3. In table 3, study number 5, there are two green bubbles are shown, but the score is just 1, this should be corrected.
**Our reply/**

We have corrected it, accordingly. "Moderate level" community involvement is attained ( Score 2).  It is Table 4 in this revised version. Thank you

**In the revised version**

Table 4. Various forms of community and extent of engagement (N =16 studies)

| 5 | Dickson,  2018 [25] |  |  | √ | √ |  |  |  |  |  |  |  | 0+1+1+0+0=2 | Moderate |
| --- | --- | --- | --- | --- | --- | --- | --- | --- | --- | --- | --- | --- | --- | --- |

Q/

In table 3, description of F form of community is missing.

**Our reply/**

It was missing in the previous edition due to formatting errors. We have corrected it, accordingly. Thank you

**In the revised version**

**Footnote of Table 4**

A. Lay persons, community leaders, volunteers,
B. Faith organization (e.g pastors),
C. Non-health Government sector such as academic partners (i.e teachers, school children/university students; pharmaceutical companies),
D. General physicians
E. Non-Governmental Organizations.
F. Politicians/activists

[NOTE: If reviewer comments were submitted as an attachment file, they will be attached to this email and accessible via the submission site. Please log into your account, locate the manuscript record, and check for the action link "View Attachments". If this link does not appear, there are no attachment files.]

Q/

While revising your submission, please upload your figure files to the Preflight Analysis and Conversion Engine (PACE) digital diagnostic tool, https://pacev2.apexcovantage.com/. PACE helps ensure that figures meet PLOS requirements. To use PACE, you must first register as a user. Registration is free. Then, login and navigate to the UPLOAD tab, where you will find detailed instructions on how to use the tool. If you encounter any issues or have any questions when using PACE, please email PLOS at figures@plos.org. Please note that Supporting Information files do not need this step.

**Our reply/**

Through PACE, we have verified figures, and each one has complied with the standards. Thank you
